# Estimation of endogenous intestinal losses of acid hydrolyzed ether extract in growing and finishing pigs using the linear regression method

**DOI:** 10.1093/tas/txab080

**Published:** 2021-05-06

**Authors:** Jesus A Acosta, R Dean Boyd, John F Patience

**Affiliations:** 1 Department of Animal Science, Iowa State University, Ames, IA 50011; 2 Hanor Company, Franklin, KY, 42134; 3 Iowa Pork Industry Center, Iowa State University, Ames, IA 50011

**Keywords:** dietary fat, energy, fat digestibility, hydrolyzed fat, swine, true total tract digestible

## Abstract

The approach of this experiment was to apply the regression method for the estimation of endogenous intestinal losses of ether extract (EEE) when pigs are fed complete diets ad libitum and using dietary levels of fat typical of those employed in commercial situations. A total of 40 gilts (PIC 337 sires × C22 or C29) were allotted to individual pens and randomly assigned to diets (8 pigs per treatment) with 5 different levels of acid hydrolyzed ether extract (AEE). The dietary treatments consisted of a corn-soybean meal diet with no added fat (L1); a corn-soy diet with 6% each of corn distiller’s dried grains with solubles (DDGS), corn germ meal, and wheat middlings (L2); the L2 diet but with 12% each of corn DDGS, corn germ meal, and wheat middlings (L3); the L2 diet plus soybean oil to equalize the NE concentration of the L2 diet with L1 (L4); and the L3 diet plus soybean oil to equalize the NE concentration of the L3 diet with L1 (L5). Pigs received feed and water ad libitum for the growing period (initial BW = 38.5 ± 1.2 kg) and the finishing period (initial BW = 73.82 ± 2.9 kg). A quadratic broken-line model was employed to estimate the response of apparent total tract digestibility (ATTD) of AEE to dietary AEE level. The average true total tract digestibility (TTTD) of AEE and endogenous losses of AEE were estimated using regression analysis of dietary AEE intake (g/kg of DM) against apparent digested AEE (g/kg of DMI). The ATTD of AEE increased in curvilinear fashion as dietary AEE level increased in growing and in finishing pigs (*P* < 0.001). This suggests an influence of EEE on the ATTD of AEE estimates. The linear regression of apparent digested AEE against dietary AEE intake (L1–L5; *P* < 0.001, *R*^2^ = 0.99 for growing pigs and *P* < 0.001, *R*^2^ = 0.99 for finishing pigs) estimated greater EEE (*P* < 0.05) and TTTD of AEE (*P* < 0.05) for growing than finishing pigs. Estimated EEE from growing pigs ranged between 18.1 and 20.2 g/kg of DMI, while TTTD of AEE ranged between 96.40% and 100.70%. In finishing pigs, EEE ranged between 21.6 and 23.8 g/kg of DMI and TTTD of AEE ranged between 91.30% and 95.25%. In conclusion, EEE under practical conditions is estimated to be 19.2 g/kg of DMI in growing and 22.7 g/kg of DMI in finishing pigs.

## INTRODUCTION

Fat supplied by the diet originates from two sources—that which is included in variable but generally small amounts in basal feed ingredients such as cereal grains, co-products, or protein sources and that which is added as a more or less pure fat source to increase the concentration of total energy (Patience, 2017); common examples of the latter include choice white grease, beef tallow, or corn oil. In addition to that provided by the diet, the contents of the intestinal tract include fat of endogenous origin, including desquamated cells, exudate from the mucosa, and bile (Clement, 1975). Apparent digestibility of EE of a simple grain-based diet is usually low—typically less than 35% (Le Goff and Noblet, 2001; Acosta et al., 2019) and increases as fats or oils are added (Adeola et al., 2013; Mendoza et al., 2014; Gutierrez et al., 2016). This increase in the apparent digestibility of dietary EE as concentrated fat sources are added to the diet is often interpreted as the consequence of their greater inherent digestibility (Li et al., 2017). The lower apparent digestibility in EE in basal ingredients may be partly due to its entrapment in the ingredient fiber matrix (Acosta et al., 2020).

However, these differences in apparent digestibility of fat in grains compared with fat sources could also reflect the impact of endogenous intestinal losses of ether extract (EEE) on the final measurement (Freeman et al., 1968). In this respect, the phenomenon is similar to that observed with the determination of amino acid digestibility. Lower EE concentrations could result in underestimates, as the impact of EEE will be more significant when total EE in the diet is low (Jørgensen et al., 1993). Therefore, estimation and correction for EEE are necessary to determine EE’s true digestibility (Zhao et al., 2017). More importantly, if this is the case, then apparent total tract digestibility (ATTD) is not an acceptable representation of the digestibility of EE and fat’s overall contribution of energy in the diet.

Regression analysis has been used to quantify EEE and to calculate true digestibility of both complete diets and ingredients (Jørgensen et al., 1993; Su et al., 2015). However, the regression method has not been tested under conditions more reflective of commercial practice; for example, most previous research has utilized purified or semi-purified diets combined with restricted feed intake (Wang et al., 2020b). To achieve precision in diet formulation, it is essential to understand if the relatively low concentration of EE found in cereal grains is of lower digestibility than EE provided by a fat-rich ingredient.

Therefore, the objective of this experiment was to determine the influence of EEE on the ATTD of fat measured in growing and finishing pigs under practical conditions (complete diets, pigs fed ad libitum, and typical levels of added fat 3%–7%) using the regression method. We hypothesized that the EEE represents a significant influence in the determination of the ATTD of fat, especially when total dietary fat levels are low.

## MATERIALS AND METHODS

All experimental procedures adhered to guidelines for the ethical and humane use of animals for research according to the Guide for the Care and Use of Laboratory Animals (FASS, 2010) and were approved by the Institutional Animal Care and Use Committee at Iowa State University (number 12-12-7478-S).

### Animals Housing and Experimental Design

This research is in continuance of a previously published study, and readers are referred to Acosta et al., (2017) for extensive experimental methods. Animal and experimental methods reported herein are provided to orient readers to the details of the study and treatment design; all analytical methods unique to these data are provided herein. Briefly, 40 crossbred gilts, the progeny of 337 sires × C22 or C29 dams (PIC Inc., Hendersonville, TN) were randomly assigned to 1 of 5 dietary treatments provided as a mash (8 pigs per treatment) for 2 periods: a growing period (initial BW 38.5 ± 0.4 kg) and a finishing period (initial BW 73.8 ± 1.1 kg). Within each period, to allow sufficient time to acclimate to the experimental diets, pigs were placed in individual pens for 21 d. They were then transferred to metabolism crates for the next 13 d. The pigs had ad libitum access to feed and water. All pigs remained on the same dietary treatment for both collection periods; however, the specific diet formulations for each treatment differed between the grower and finisher period to reflect their differing nutrition requirements (Table 1).

**Table 1.** Composition of experimental diets, as fed basis^*^

	Growing pigs					Finishing pigs				
Item	L1	L2	L3	L4	L5	L1	L2	L3	L4	L5
Ingredient, %										
Corn	72.39	58.25	44.06	56.46	40.58	79.61	65.45	51.26	63.67	47.68
Soybean meal	23.90	20.27	16.64	20.40	16.89	16.95	13.31	9.68	13.44	9.93
Corn DDGS	–	6.00	12.00	6.00	12.00	–	6.00	12.00	6.00	12.00
Corn germ meal	–	6.00	12.00	6.00	12.00	–	6.00	12.00	6.00	12.00
Wheat middlings	–	6.00	12.00	6.00	12.00	–	6.00	12.00	6.00	12.00
Soybean oil	–	–	–	1.66	3.32	–	–	–	1.66	3.32
L-lys HCl	0.30	0.30	0.30	0.30	0.30	0.30	0.30	0.30	0.30	0.30
DL-methionine	0.06	0.01	–	0.01	–	0.03	–	–	–	–
L-threonine	0.08	0.06	0.05	0.06	0.05	0.07	0.06	0.04	0.06	0.05
Monocalcium phosphate	0.91	0.62	0.33	0.63	0.34	0.80	0.51	0.22	0.52	0.23
Limestone	1.15	1.28	1.41	1.27	1.40	1.03	1.16	1.28	1.15	1.28
Salt	0.50	0.50	0.50	0.50	0.50	0.50	0.50	0.50	0.50	0.50
Vitamin premix^†^	0.16	0.16	0.16	0.16	0.16	0.16	0.16	0.16	0.16	0.16
Trace mineral premix^‡^	0.15	0.15	0.15	0.15	0.15	0.15	0.15	0.15	0.15	0.15
Titanium dioxide	0.40	0.40	0.40	0.40	0.40	0.40	0.40	0.40	0.40	0.40
Total	100.00	100.00	100.00	100.00	100.00	100.00	100.00	100.00	100.00	100.00
Analyzed chemical composition										
DM, %	88.54	88.36	89.16	88.70	89.19	88.31	88.47	89.25	88.73	89.22
GE, Mcal/kg	3.84	3.90	3.94	3.99	4.12	3.78	3.86	3.97	3.96	4.12
CP, %	18.15	19.24	20.20	18.94	19.97	14.78	15.95	17.46	15.99	17.21
NDF, %	5.60	10.40	15.30	10.30	15.10	5.70	10.50	15.30	10.40	15.10
AEE^||^, %	2.91	3.30	3.79	4.89	7.01	3.02	3.51	3.89	5.11	7.10
Calculated chemical composition										
Lys, %	1.16	1.15	1.13	1.17	1.13	0.91	0.94	0.94	0.93	0.95
SID^$^ Lys, %	1.03	0.99	0.93	1.00	0.93	0.80	0.80	0.76	0.78	0.77
NE, Mcal/kg	2.43	2.35	2.27	2.43	2.43	2.49	2.41	2.32	2.49	2.48
Ca, %	0.69	0.69	0.69	0.69	0.69	0.60	0.60	0.60	0.60	0.60
STTD^$^ P, %	0.32	0.32	0.32	0.32	0.32	0.28	0.28	0.28	0.28	0.28

*L1 = basal diet with no added fat; L2 = L1 with 6% each of corn, DDGS, corn germ meal, and wheat middlings and energy allowed to float; L3 = L1 with 12% each of corn, DDGS, corn germ meal, and wheat middlings and energy allowed to float; L4 = L2 diet plus soybean oil to equalize NE to that of L1; L5 = L3 plus soybean oil to equalize NE to that of L1.

^†^Provided per kg of diet: 4,900 IU of vitamin A; 560 IU of vitamin D_3_; 40 IU of vitamin E; 2.4 mg of menadione (to provide vitamin K); 39 μg of vitamin B_12_; 9 mg of riboflavin; 22 mg of d-pantothenic acid; and 45 mg of niacin.

^‡^Provided per kg of diet: 165 mg of Fe (ferrous sulfate); 165 mg of Zn (zinc sulfate); 39 mg of Mn (manganese sulfate); 2 mg of Cu (copper sulfate); 0.3 ppm of I (calcium iodate); and 0.3 ppm of Se (sodium selenite).

^||^AEE = acid hydrolyzed ether extract.

^$^SID = standardized ileal digestible.

### Dietary Treatments

Diets for the growing and for the finishing period were manufactured using commercial sources of ingredients to achieve five different acid hydrolyzed ether extract (AEE) levels. The control diet consisted of a typical corn-soybean meal formula with no added fat (L1; Table 1); the L2 and L4 diets contained 6% each of corn distiller’s dried grains with solubles (DDGS), corn germ meal, and wheat middlings; and the L3 and L5 diets contained 12% each of corn DDGS, corn germ meal, and wheat middlings. The net energy content of diets L2 and L3 was allowed to vary, meaning no fat was added and as a result, the NE content declined. Soybean oil was added to the L4 and L5 diets to equalize the NE content of L1. NE was calculated according to equation (1)–(7) (NRC, 2012) using ingredient assay results as previously described (Acosta et al., 2017). The main difference between L2 and L4 was the added fat, similar to the difference between L3 and L5. These diet formulations provide a sufficient range in AEE to achieve the objectives of the experiment. These formulations, differing in coproduct ingredients, represented typical ranges in commercial diet composition, and by lowering NE, provided a foundation to add two levels of added fat. To avoid confounding of experiment outcomes, as many ingredients as possible were maintained at the same level across all diets within growth phase (L1 to L5). Any potential effect of fiber level on fat digestibility was addressed by having added fat, and no added fat, within each NDF level. In this way, as much as possible, the results of the experiment in terms of fat digestibility would be due to differences in fat content. The composition of the diets also reflected commercial diets, key to achieving the objective of the experiment.

Amino acids, phosphorous, and calcium levels were set to meet or exceed the NRC (2012) requirements for gilts for both growing and finishing periods. Additionally, TiO_2_ was included at 0.4% as an indigestible marker to facilitate calculation of diet and fat digestibility.

### Data and Samples

A total of 10 samples of each diet were randomly collected at the feed mill at the time of mixing and then thoroughly homogenized and carefully subsampled. After 3 d adaptation of pigs in metabolism crates, fresh fecal samples were obtained twice daily during d 4–6 and d 11–13, resulting in two collections per pig. Feces were placed in pre-labeled plastic bags and stored at −20°C until further processed. Once collected, fecal samples were homogenized and subsampled. Then, subsamples were dried in an oven at 105°C and ground through a 1 mm screen in a Wiley grinder (Model ED-5, Thomas Scientific Inc., Swedesboro, NJ). Feed samples were ground through a 1 mm screen in a Retsch grinder (Model ZM1, Retsch Inc., Newton, PA). Dried fecal and feed samples were kept in plastic bags and stored in desiccator cabinets until chemical assays were performed.

Samples of feed and feces were analyzed to determine DM concentration (method 930.15; AOAC, 2007), AEE was assayed using a SoxCap hydrolyzer (model SC 247) and a Soxtec fat extractor (model 255; Foss, Eden Prairie, MN; method 968; AOAC, 2007), and TiO_2_ determined using a Synergy 4 spectrophotometer (BioTek, Winooski, VT) according to the method of Leone (1973).

### Calculations

The results of the assays of the feces within each collection period (d 4–6 and d 11–13) were calculated separately and then combined in the statistical model (see below). ATTD of AEE was calculated using the equation proposed by Oresanya et al. (2008): ATTD of AEE, % = 100 − [100 × (% TiO_2_ in feed/% TiO_2_ in feces) × (concentration of component in feces/concentration of component in feed)].

Apparent digested AEE (g/kg DMI) was calculated by multiplying dietary AEE intake (g/kg of DM) times the ATTD of AEE (%). The average true total tract digestibility (TTTD) of AEE and endogenous losses of AEE were estimated using regression analysis of dietary AEE intake (g/kg of DM) against apparent digested AEE (g/kg of DMI) according to Zhao et al. (2017). The TTTD for each observation was calculated using the following equation (Jørgensen et al., 1993): TTTD of AEE, % = {1 − [(dietary AEE intake − apparent digested AEE) − EEE]}/dietary AEE intake) × 100, in which dietary AEE intake is g/kg of DM, apparent digested AEE is in g/kg DMI, EEE is in g/kg of DMI.

### Statistical Analysis

The NLMIXED procedure of SAS was used to fit a quadratic broken-line model (as described by Robbins et al., 2006) between the ATTD of AEE and dietary AEE intake. The REG procedure of SAS was used to fit a linear model between apparent digested AEE and dietary AEE intake. The EEE AEE for growing and finishing pigs were estimated as the intercept of the linear equation derived from the regression between apparent digested AEE and dietary AEE intake. The intercepts were compared by the overlapping of the 95% confidence intervals. The ATTD and TTTD were analyzed using the MIXED procedure of SAS with AEE level as fixed effect. The effect of collection was not significant and therefore was removed from the model. Since they were housed individually, each pig represented the experimental unit for all analyses. Probability values less than 0.05 were considered significant.

## RESULTS AND DISCUSSION

All pigs remained healthy during the growing and the finishing period. They did not show any sign of disease or off-feeding events, and there was no mortality nor any need for medical treatments.

**Figure 1.**
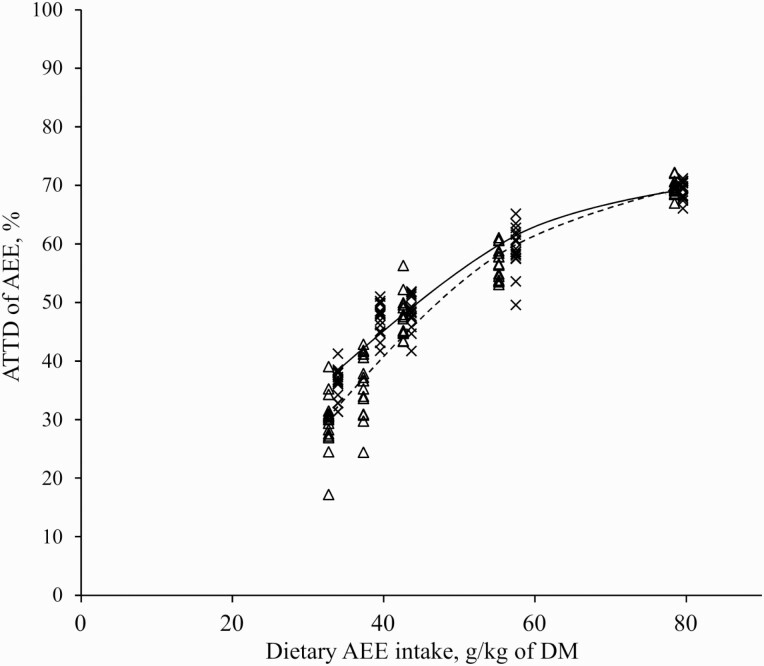
The quadratic broken-line: *y* = *L* + *U* × (*R* − *x*) × (*R* − *x*), where (*R* − *x*) is zero at values of *x* > *R*, fitted to the ATTD response to dietary AEE intake for growing pigs (--∆--) and (―×―) finishing pigs.

**Figure 2.**
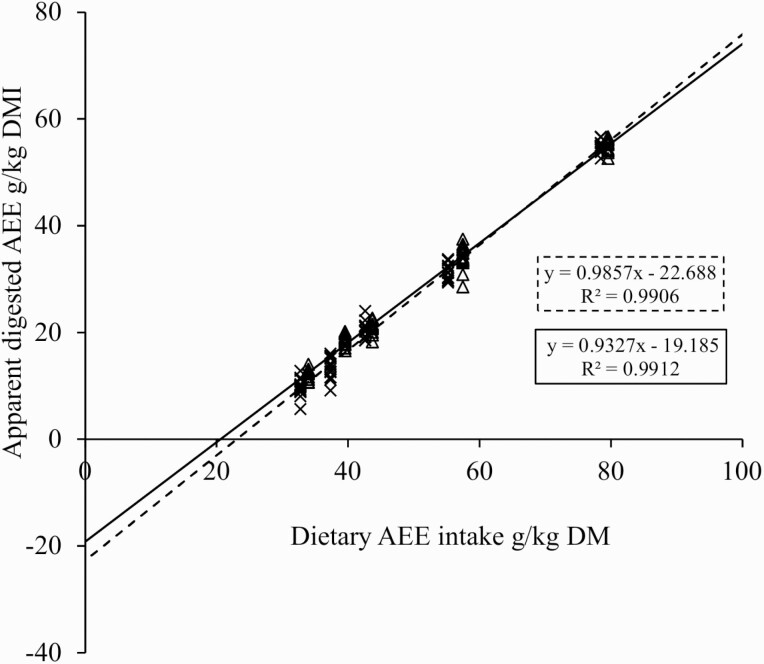
Estimation of endogenous losses of AEE by regression of dietary AEE intake against apparent digested AEE in growing pigs (―×―; *P* < 0.001) and finishing pigs (--∆--; *P* < 0.001).

In both the growing and the finishing periods, the ATTD of AEE increased in a curvilinear (quadratic broken-line) fashion for both growing and finishing pigs as the intake of AEE increased (Figure 1). A linear relationship between apparent digested AEE and dietary AEE intake was also established for the growing and finishing periods. Both linear regression equations showed very high coefficients of determination (*P* < 0.001, *R*^2^ = 0.99 and *P* < 0.001, *R*^2^ = 0.99 for growing and finishing pigs, respectively; Figure 2). Additionally, TTTD of AEE was greater for growing than for finishing pigs (98.6% vs. 93.27%; *P* < 0.05).

Estimated EEE was greater for growing than for finishing pigs (22.7 vs. 19.2 g/kg DMI; *P* < 0.05; Table 2). The estimated 95% confidence interval of EEE for growing pigs ranged between 21.6 and 23.8 g/kg of DMI. For finishing pigs, the 95% confidence interval of EEE ranged between 18.1 and 20.2 g/kg of DMI. There was no relationship between dietary AEE level and TTTD in growing (*P* = 0.989; Table 3) or finishing pigs (*P* = 0.899).

**Table 2.** Estimated intestinal EEE of AEE for growing and finishing pigs fed complete diets ad libitum^*^

	EEE, g/kg of DMI			
Item	Estimate	SE	95% CI	
Growing pigs	22.7^a^	0.6	21.6	23.8
Finishing pigs	19.2^b^	0.5	18.1	20.2

^*^Data were analyzed with the REG procedure of SAS using dietary AEE intake (g/kg of DM) regressed against apparent digested AEE (g/kg of DMI). Estimated EEE is derived from the apparent digested AEE at zero intake (Figure 2).

^a,b^Within a column, values lacking a common superscript are different (*P* < 0.05).

**Table 3.** The relationship between the TTTD of AEE determined in the growing and the finishing period

	Level of AEE^*^						
Item	L1	L2	L3	L4	L5	SEM	*P*-value
Growing pigs							
Dietary AEE level DM, %	3.28	3.74	4.26	5.52	7.85	–	–
ATTD of AEE, %	29.6^a^	36.0^b^	47.5^c^	56.8^d^	69.8^e^	1.0	<0.001
TTTD of AEE, %	98.9	96.7	100.7	97.8	98.6	0.9	0.989
Finishing pigs							
Dietary AEE level DM, %	3.40	3.96	4.37	5.75	7.96	–	–
ATTD of AEE, %	36.0^a^	47.2^b^	48.2^b^	59.2^c^	69.4^d^	1.0	<0.001
TTTD of AEE, %	92.5	95.6	92.1	92.6	93.5	0.7	0.899

^*^L1 = basal diet with no added fat; L2 = L1 with 6% each of corn, DDGS, corn germ meal, and wheat middlings and energy allowed to float; L3 = L1 with 12% each of corn, DDGS, corn germ meal, and wheat middlings and energy allowed to float; L4 = L2 diet plus soybean oil to equalize NE to that of L1; L5 = L3 plus soybean oil to equalize NE to that of L1.

^a–e^Means within a row with different superscripts differ (*P* ≤ 0.05).

There are at least three methods for the estimation or measurement of EEE. First, by using radiolabeled dietary fat, endogenous and dietary fat can be separated (Freeman et al., 1968). Second, using a fat-“free” diet, basal EEE can be estimated in the same manner as for amino acids (Stein et al., 2007; Wang et al., 2020a). Third, the apparent digested AEE can be determined using a linear regression method to estimate total EEE; this was the approch used in this study and in previous research (Gutierrez et al., 2016; Zhou et al., 2017). A relevant advantage of the linear regression method is that it can be easily applied under more practical circumstances using formulations reflective of commercial diets.

Two conditions are necessary to estimate endogenous secretions of fat through the regression method used herein. The first one involves a typical curvilinear function between dietary fat level and fat digestibility. It is necessary to assume an effect of endogenous secretions on digestibility values (Stein et al., 2007). Likewise, linear regression with high coefficients of determination is required to estimate EEE (Kil et al., 2010). It is relevant to mention that the regression method is a mathematical estimation. The intercept attributes a proportion of the total fat excreted to endogenous secretions (Zhao et al., 2017), while the slope represents the remaining proportion. The data from this experiment met both requirements.

The current experiment confirmed the influence of EEE on the ATTD of AEE described in previous studies (Kil et al., 2010; Zhou et al., 2017). Moreover, these results suggest that EEE plays a significant role in ATTD estimations at the dietary AEE levels (3%–8%) used in most commercial diets. Thus, correction for EEE is essential when comparing apparent digestibilities among these different levels of fat.

This experiment suggested that the EEE from complete diets fed ad libitum was estimated to be ~21 g/kg of DMI. Across studies, different EEE values along the total tract have been reported. By feeding growing pigs with purified diets and using the regression method, some studies have reported values of EEE of 4.41 g/kg of DMI by adding soybean oil (fat levels from 0.5% to 3%; Jørgensen et al., 1993). Kil et al. (2010) reported EEE values of 3.77 and 12.08 g/kg DMI, respectively, when adding corn oil (fat levels from 1.3% to 6.9%) or corn germ meal (fat levels from 3.03% to 9.74%), respectively. Kim et al. (2013) feeding semi-purified diets reported EEE levels ranging from −0.11 to 6.51 g/kg of DMI in diets with different corn ingredients (fat levels from 1.3% to 7.4%) and 4.85 g/kg of DMI in diets with full-fat soybeans (fat levels from 1.3% to 7.9%).

Higher estimates of EEE have been reported in pigs fed complete diets. Adams and Jensen (1985) reported EEE of 8.7 g/kg of DMI in pigs fed complete diets with increasing fat from sunflower seeds (fat levels from 12.7% to 27.7%). Likewise, Su et al. (2015) reported EEE of 10.8 and 14.0g/kg of DMI by adding palm oil and soybean oil, respectively (fat levels from 2.9% to 12.7%). Gutierrez et al. (2016) reported EEE of 13.6 g/kg of DMI by adding reduced-oil DDGS, and soybean oil (fat levels from 4.4% to 10.1%). Zhao et al. (2017) estimated an EEE of 13.8 g/kg of DMI by adding cottonseed oil (fat levels from 2.6% to 12.0%). Estimations closer to those reported in the current study have also been observed. Jørgensen and Fernandez (2000) reported EEE of 22.4 g/kg of DMI using barley–soybean meal diets with high levels (5.0% to 30.0%) of extracted fats, while Zhou et al. (2017) estimated EEE of 23.0 and 23.9 g/kg of DMI in diets with canola press-cake (fat levels 1.6% to 7.3%) and canola oil (fat levels 1.6% to 5.6%), respectively. No EEE values in pigs fed complete diets ad libitum were found in the literature.

The differing estimates if EEE among fat sources described above, even within a study, is perplexing. We started this study assuming that EEE would be independent of fat source. However, this assumption may be flawed as it is entirely possible that EEE is impacted by fat source, just as true ileal digestibility of amino acids is dependent on source, considering both basal and specific endogenous losses (Adeola et al., 2016). However, the relative dearth of information on endogenous fat secretions provides no proof one way or the other on this topic. The limited data that are available suggest that there is both basal and specific endogenous losses of fat, and that specific losses differ based on fat source. Clearly, more research is needed to address this important question.

Differences between the study reported herein and those reported in the literature probably explain the variable outcomes. In the current study, complete diets instead of purified diets were used, and perhaps even more critically, pigs were fed ad libitum instead of restricted. Indeed, the current experiment’s importance arises from the fact that it was conducted using these conditions and satisfied the principles to calculate EEE using the regression method. The linear relationship illustrated in Figure 2 shows an evident alignment between the dietary AEE levels and the apparent digested AEE. This relationship suggests that the pigs digested the AEE with differing efficiencies as dietary AEE increased. By regressing AEE intake against digestible AEE, a linear response becomes apparent and takes out the substantial impact of both source and level of fat in the diet.

Also, the results of the TTTD of AEE in the current experiment suggests that AEE is highly digested along the entire intestinal tract (>90%). High true digestibilities are commonly observed after correction by EEE (Gutierrez et al., 2016). The classical approach of using EEE is to assign digestible values to dietary sources (Chen et al., 2019; Wang et al., 2020b). However, knowing EEE becomes even more critical when researchers seek to model digestion and absorption of energy and nutrients in pigs (Birkett and de Lange, 2001; Strathe et al., 2008). Based on the data generated herein, EEE can represent about 189 kcal/kg of DMI of the pig’s maintenance energy (assuming EEE of 21 g/kg DMI and 9,000 kcal/kg of AEE). If accurate, EEE represents an estimable energy expenditure and can be applied in future energy models.

In conclusion, the EEE using the regression method in complete diets fed ad libitum were estimated to be 19.2 g/kg of DMI in growing and 22.7g/kg of DMI in finishing pigs. Results indicate that the EEE exerts a significant influence on the apparent digestibility at the levels commonly used in the swine industry. Therefore, evaluation of the digestibility of fat should be interpreted after the correction for endogenous fat secretions. Further research is needed to describe and allocate the contribution of EEE to the pig’s maintenance energy requirements and to further elucidate the factors that impact EEE.
